# Endoplasmic Reticulum Stress Induces Different Molecular Structural Alterations in Human Dilated and Ischemic Cardiomyopathy

**DOI:** 10.1371/journal.pone.0107635

**Published:** 2014-09-16

**Authors:** Ana Ortega, Esther Roselló-Lletí, Estefanía Tarazón, Maria Micaela Molina-Navarro, Luis Martínez-Dolz, José Ramón González-Juanatey, Francisca Lago, Jose David Montoro-Mateos, Antonio Salvador, Miguel Rivera, Manuel Portolés

**Affiliations:** 1 Cardiocirculatory Unit, Health Research Institute of La Fe University Hospital, Valencia, Spain; 2 Heart Failure and Transplantation Unit, Cardiology Department, La Fe University Hospital, Valencia, Spain; 3 Cellular and Molecular Cardiology Research Unit, Department of Cardiology and Institute of Biomedical Research, University Clinical Hospital, Santiago de Compostela, Spain; UCL Institute of Child Health, United Kingdom

## Abstract

**Background:**

The endoplasmic reticulum (ER) is a multifunctional organelle responsible for the synthesis and folding of proteins as well as for signalling and calcium storage, that has been linked to the contraction-relaxation process. Perturbations of its homeostasis activate a stress response in diseases such as heart failure (HF). To elucidate the alterations in ER molecular components, we analyze the levels of ER stress and structure proteins in human dilated (DCM) and ischemic (ICM) cardiomyopathies, and its relationship with patient's functional status.

**Methods and Results:**

We examined 52 explanted human hearts from DCM (n = 21) and ICM (n = 21) subjects and 10 non-failing hearts as controls. Our results showed specific changes in stress (IRE1, *p*<0.05; p-IRE1, *p*<0.05) and structural (Reticulon 1, *p*<0.01) protein levels. The stress proteins GRP78, XBP1 and ATF6 as well as the structural proteins RRBP1, kinectin, and Nogo A and B, were upregulated in both DCM and ICM patients. Immunofluorescence results were concordant with quantified Western blot levels. Moreover, we show a novel relationship between stress and structural proteins. RRBP1, involved in procollagen synthesis and remodeling, was related with left ventricular function.

**Conclusions:**

In the present study, we report the existence of alterations in ER stress response and shaping proteins. We show a plausible effect of the ER stress on ER structure in a suitable sample of DCM and ICM subjects. Patients with higher values of RRBP1 had worse left ventricular function.

## Introduction

Heart failure (HF) is a syndrome with high morbidity and mortality in industrialized countries. This syndrome is usually associated with left ventricular (LV) systolic dysfunction, although diastolic impairment at rest is a frequent if not universal accompaniment [1]. HF is the end stage of many heart diseases [2] including dilated (DCM) and ischemic cardiomyopathies (ICM).

Several studies have associated HF with cellular and molecular alterations in cardiac tissue, including abnormal calcium handling [3]; changes in mitochondria [4], [5], nuclear components [6]–[8], cytoskeletal proteins [9], [10], and also alterations in the endoplasmic reticulum (ER) [11]. In particular, disruption of ER homeostasis has been linked to several processes of cardiovascular diseases including ischemia/reperfusion injury [12], DCM [13] and HF [14].

The ER is the primary site of secretory protein synthesis and maturation, calcium storage, and lipid biosynthesis. Various factors can disrupt ER homeostasis and alter its functions (ER stress), provoking the accumulation of unfolded and misfolded proteins in the ER lumen and leading to cellular and pathological dysfunctions. ER stress triggers a cellular response in which different pathways are implicated in maintaining ER homeostasis through attenuation of protein synthesis, transcriptional induction of ER chaperone genes and ER-associated degradation component genes, and finally, the induction of apoptosis to safely ensure the survival of the whole organism [11], [15]. In all of these pathways, lumen and transmembrane proteins such as inositol-requiring protein-1 (IRE1) and glucose-related protein 78 (GRP78), and transcription factors such as X-box binding protein 1 (XBP1), activating transcription factor 6 (ATF6), ATF4 and eukaryotic initiation factor 2 alpha (eIF2α), act together to produce the ER stress response [16].

ER stress promotes the synthesis and expression of molecules implicated in the unfolded protein response (UPR), and this alteration could also occur at the structural level of the ER, leading to changes in the expression of proteins (e.g., scaffolding proteins and curvature stabilizing proteins) that stabilize the multiple folds that shapes the ER. These changes could impair the function of the ER and have undesirable consequences.

In this study, we hypothesize that patients with HF may display changes in proteins involved in the ER stress response and that these changes could differ between the DCM and ICM pathologies. Furthermore, we aim to establish a new link between these alterations and changes of structural proteins of the ER. Therefore, our objective was to analyze levels of highly representative ER stress proteins and ER structural proteins in LV tissue from patients with DCM and ICM. Moreover, we studied the relationship between these proteins and changes in LV function.

## Materials and Methods

### Ethics statement

All patients gave written informed consent to participate in the study. The project was approved by the Ethics Committee (Biomedical Investigation Ethics Committee of La Fe University Hospital of Valencia, Spain) and conducted in accordance with the principles of the current version of the Declaration of Helsinki [17].

### Collection of samples

A total of 42 pathological human hearts were obtained from 21 patients with DCM and 21 with ICM undergoing cardiac transplantation. Clinical history, ECG, Doppler echocardiography, hemodynamic studies, and coronary angiography data were available on all of these patients. All patients were functionally classified according to the New York Heart Association (NYHA) criteria and were receiving medical treatment following the guidelines of the European Society of Cardiology [18]. Non-ischemic DCM was diagnosed when patients had intact coronary arteries on coronary angiography and LV systolic dysfunction [ejection fraction (EF) <40%] with a dilated non-hypertrophic left ventricle [LV end-diastolic diameter (LVEDD) >55 mm] on echocardiography. Furthermore, patients showed no more than mild valvular disease.

As control (CNT) samples, 10 non-diseased donor hearts were used. The cause of death was cerebrovascular accident or motor vehicle accident. All donors had normal LV function and no history of myocardial disease. The hearts were initially considered for cardiac transplantation but were subsequently deemed unsuitable either because of blood type or size incompatibility.

LV samples were taken from near the apex of the left ventricle and were flushed with 0.9% NaCl and stored at 4°C for a mean of 4.4 h from the time of coronary circulation loss. Samples were then placed at −80°C until the protein extraction procedure.

### Homogenization of samples and protein determination

Twenty-five milligrams of frozen left ventricle was transferred into Lysing Matrix D tubes designed for use with the FastPrep-24 homogenizer (MP Biomedicals, USA) in a total protein extraction buffer (2% SDS, 10 mM EDTA, 6 mM Tris–HCl, pH 7.4) with protease inhibitors (25 µg/mL aprotinin and 10 µg/mL leupeptin). The homogenates were centrifuged at 16100× *g*, and the supernatant was aliquoted. The protein content of the aliquot was determined using Peterson's modification of the micro Lowry method with bovine serum albumin (BSA) as the standard [19].

### Polyacrylamide gel electrophoresis and Western blot analysis

Protein samples for detection of IRE1, IRE1 phosphorylated (p-IRE1), GRP78, XBP1, ATF6, ATF4, eIF2α phosphorylated (p-eIF2α), Reticulon 1, ribosomal receptor-binding protein 1 (RRBP1), kinectin, and cytoskeleton-associated protein 4 (CKAP4) were separated using Bis-Tris Midi gel electrophoresis with 4–12% polyacrylamide. Tris-acetate 3–8% polyacrylamide gel was used for Nogo (Reticulon 4) A and B isoforms separation. After electrophoresis, the proteins were transferred from the gel to a PVDF membrane using the iBlot Dry Blotting System (Invitrogen Ltd, UK) for Western blot analyses. The membranes were blocked overnight at 4°C with 1% BSA in Tris buffer solution containing 0.05% Tween 20 and, after blocking, were incubated for 2 h with the primary antibody in the same buffer. The primary detection antibodies used were anti-IRE1 mouse monoclonal antibody (1∶400), anti-p-IRE1 rabbit monoclonal antibody (1∶200), anti-GRP78 rabbit polyclonal antibody (1∶600), anti-XBP1 mouse monoclonal antibody (1∶400), anti-ATF6 rabbit polyclonal antibody (1∶1000), anti-ATF4 mouse monoclonal antibody (1∶400), anti-p-eIF2α rabbit monoclonal antibody (1∶250), anti-Reticulon 1 rabbit polyclonal antibody (1∶500), anti-RRBP1 rabbit polyclonal antibody (1∶250), anti-kinectin rabbit polyclonal antibody (1∶500), anti-CKAP4 rabbit polyclonal antibody (1∶1000), and anti-Nogo A+B rabbit polyclonal antibody (1∶250). Anti-GAPDH mouse monoclonal antibody (1∶1000) was used as a loading control. All antibodies were from Abcam (Cambridge, UK) except for p-IRE1 and p-eIF2α that were from EMD Millipore Corporation (Billerica, MA, USA).

The bands were then visualized using an acid phosphatase-conjugated secondary antibody and nitro blue tetrazolium/5-bromo-4-chloro-3-indolyl phosphate (NBT/BCIP, Sigma-Aldrich, St. Louis, USA) substrate system. Finally, the bands were digitalized using an image analyzer (DNR Bio-Imagining Systems, Israel) and quantified with the GelQuant Pro (v. 12.2) program.

### Fluorescence microscopy analysis

Cardiac LV muscle samples were fixed in 4% formalin, embedded in paraffin blocks and sectioned into 5 µm using a microtome Microm HM 340E (Thermo Fisher Scientific Inc., Walldorf, Germany). Sections were transferred to glass slides and were kept at 60°C overnight, deparaffinized with xylol followed by washing in 100%, 96%, 80%, and 70% ethanol. Samples were then fixed in pure methanol for 4 min at −20°C. Sections were then blocked with PBS containing 1% BSA for 15 min at room temperature (RT). After blocking, sections were incubated for 2 h at RT with the primary antibodies for RRBP1 (1∶200 dilution), kinectin (1∶150 dilution), and Nogo A+B (1∶200 dilution) (described in preceding section) in the same buffer solution and then with Alexa-conjugated secondary antibody (Invitrogen, USA) for 1 h at RT [20]. Then, sections were rinsed in PBS, mounted in Vectashield with DAPI for identifying nuclei (Vector, Burlingame, CA, USA), and observed with an Olympus BX50 fluorescence microscope (Tokyo, Japan). Finally, the images were processed using ImageJ (v. 1.46 r; National Institutes of Health, Bethesda, MD, USA) software.

### Statistical methods

Data are presented as mean values ± standard deviation. The Kolmogorov–Smirnov test was used to analyze the distribution of variables. Comparisons of clinical characteristics were performed using the Student's *t*-test for continuous variables and the Fisher's exact test for discrete variables. Comparisons of protein expression levels between different groups were performed using the Student's *t*-test for variables with a normal distribution and the Mann–Whitney U test for variables with non-normal distribution. RRBP1 levels exhibited a non-normal distribution and were log transformed (and proved to be normalized) before parametric correlation analysis. Finally, Pearson's correlation coefficient was performed to analyze the association between variables. Significance was assumed at *p*<0.05. All statistical analyses were performed using SPSS software v. 20 for Windows (IBM SPSS Inc., Chicago, IL, USA).

## Results

### Clinical characteristics of patients

We analyzed 52 human hearts, including 42 explanted human hearts from patients diagnosed with HF (21 DCM and 21 ICM) undergoing cardiac transplantation and 10 non-diseased donor hearts as CNT samples. Most of the patients were men (79%) with a mean age of 53±11 years, a mean NYHA functional classification of III–IV, and previous diagnosis of significant comorbidities including hypertension, hypercholesterolemia, and diabetes mellitus. The clinical characteristics and echocardiographic parameters of HF patients according to etiology are shown in Table 1. The DCM group was significantly younger than ICM patients (*p*<0.05) and showed reduced levels of total cholesterol (*p*<0.01). Significant differences were also found in LV end-systolic diameter (LVESD) (*p*<0.05), LVEDD (*p*<0.05), and LV mass index (LVMI) (*p*<0.05), with higher values in the DCM group. The CNT group was comprised mainly of men (70%) with a mean age of 46±18 years.

**Table 1:** Clinical characteristics of patients according to HF etiology.

	DCM (n = 21)	ICM (n = 21)
Age (years)	49±13	57±7*
Gender male (%)	79	79
NYHA class	3.4±0.4	3.3±0.5
BMI (kg/m^2^)	25±6	25±3
Hemoglobin (mg/dL)	14±2	13±2
Hematocrit (%)	41±6	37±7
Total cholesterol (mg/dL)	142±42	185±40**
Prior hypertension (%)	25	46
Prior smoking (%)	63	75
Diabetes mellitus (%)	15	29
EF (%)	22±8	22±8
FS (%)	12±4	13±6
LVESD (mm)	65±11	56±9*
LVEDD (mm)	74±11	64±8*
Left ventricle mass index (g/cm^2^)	204±64	147±46*

DCM, dilated cardiomyopathy; ICM, ischemic cardiomyopathy; NYHA class, New York Heart Association class; BMI, body mass index; EF, ejection fraction; FS, fractional shortening; LVESD, left ventricular end-systolic diameter; LVEDD, left ventricular end-diastolic diameter.

*p<0.05;

**p<0.01.

### HF etiology related changes in ER stress protein levels

**Figure 1:**
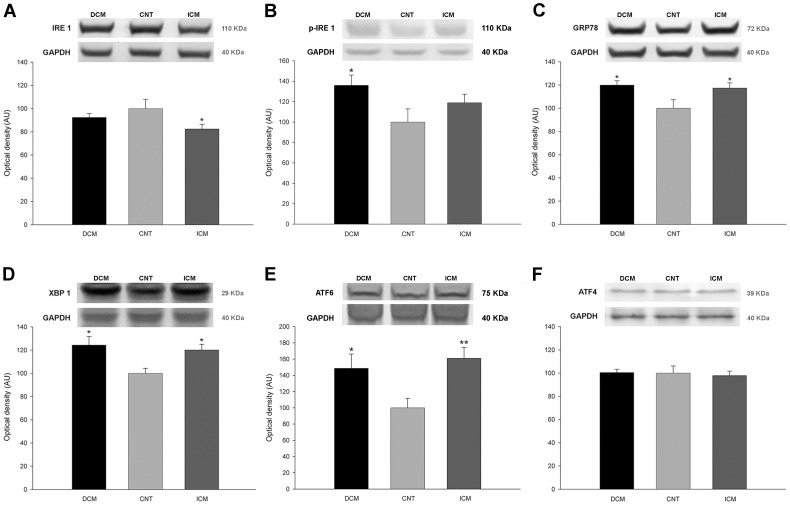
Protein expression levels of endoplasmic reticulum stress proteins in dilated (DCM) and ischemic (ICM) cardiomyopathy and control (CNT) groups. (**A**) Inositol-requiring protein-1 (IRE1). (**B**) Inositol-requiring protein-1 phosphorylated (p-IRE1) (**C**) Glucose-related protein 78 (GRP78). (**D**) X-box binding protein 1 (XBP1). (**E**) Activating transcription factor 6 (ATF6) (**F**) Activating transcription factor 4 (ATF4). The values are normalized to GAPDH and finally to the CNT group. Values from the CNT group are set to 100. The data are expressed as means ± standard error of the mean (SEM) in optical density, arbitrary units (AU), of six independent experiments. *p<0.05, **p<0.01 *vs.* the CNT group.

We focused our study on the stress proteins IRE1, p-IRE1, GRP78, XBP1, ATF6, ATF4 and p-eIF2α that are highly representative in the activation of the UPR during ER stress process. When we compared stress protein levels between the pathological groups and our 10 CNT hearts, we found that IRE1 was significantly reduced in ICM [82±19 *vs.* 100±23 arbitrary units (AU), *p*<0.05] (Fig. 1A), while p-IRE1 was upregulated in DCM patients (136±29 *vs.* 100±34 AU, *p*<0.05) (Fig. 1B). GRP78, XBP1 and ATF6 were significantly increased in both DCM (120±19 *vs.* 100±23 AU, *p*<0.05, 124±33 *vs.* 100±14 AU, *p*<0.05, and 149±53 *vs* 100±31 AU, *p*<0.05, respectively) and ICM samples (117±21 *vs.* 100±23 AU, *p*<0.05, 120±22 *vs.* 100±14 AU, and 161±40 *vs* 100±31 AU, *p*<0.01, respectively) (Fig. 1C, D and E). The levels of ATF4 (Fig. 1F) and p-eIF2α did not show any changes in both pathologies.

### Levels of ER structural proteins according to HF etiology

**Figure 2:**
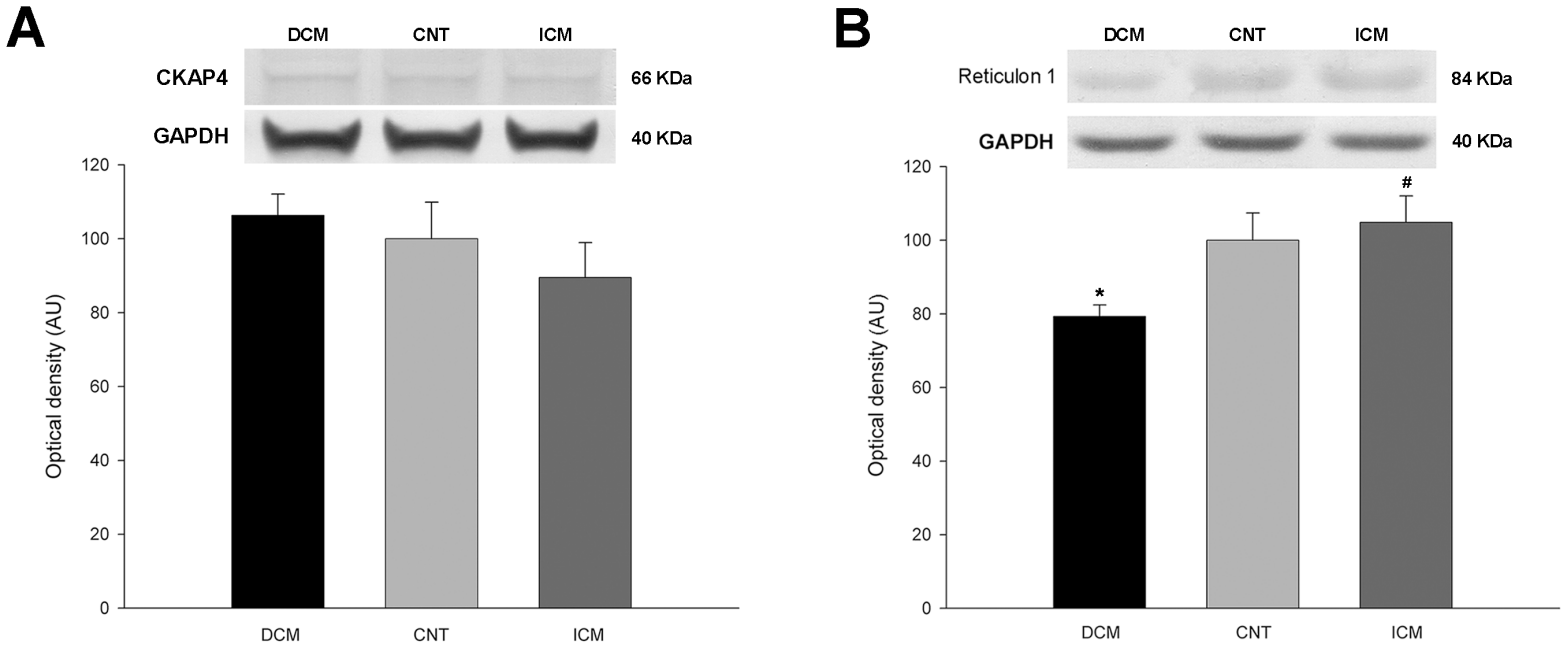
Western blot analysis of structural proteins of the endoplasmic reticulum in dilated (DCM) and ischemic (ICM) cardiomyopathy and control (CNT) groups. (**A**) Cytoskeleton-associated protein 4 (CKAP4). (**B**) Reticulon 1. The values are normalized to GAPDH and finally to the CNT group. Values from the CNT group are set to 100. The data are expressed as means ± standard error of the mean (SEM) in optical density, arbitrary units (AU), of two independent experiments. *p<0.01 *vs.* the CNT group. #*p*<0.01 DCM *vs.* the ICM group.

We analyzed the protein levels of the structural proteins Reticulon 1, RRBP1, kinectin, CKAP4 and two Nogo (Reticulon 4) isoforms (A and B); which are important proteins shaping the ER. Western blot experiments revealed significant alterations in the amount of distinct structural proteins of the ER organelle. However, we found no statistically significant differences between levels of CKAP4 protein in the two HF etiologies compared to CNT group (Fig. 2A). Reticulon1 showed protein changes according to HF etiology. Whereas this protein was significantly reduced in the DCM group compared with levels in the CNT group (79±13 *vs.* 100±17 AU, *p*<0.01), we observed no significant alterations in the ICM group. On the other hand, when we compared the levels of Reticulon 1 in the DCM and ICM groups, we found significant differences (79±13 *vs.* 105±31 AU, *p*<0.01), (Fig. 2B).

**Figure 3:**
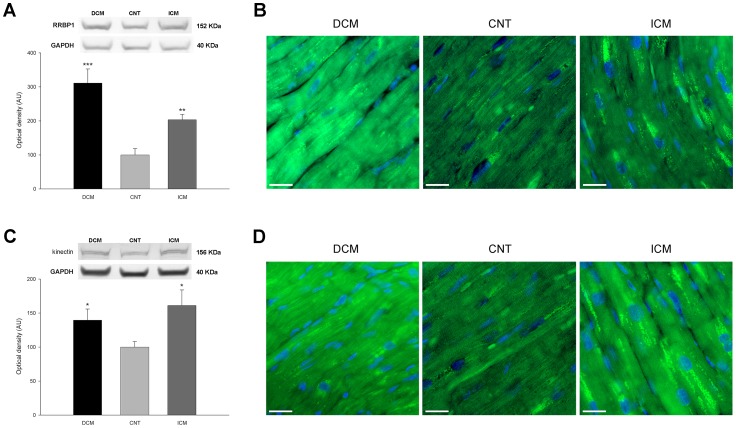
Western blot and immunofluorescence studies of cell distribution of some endoplasmic reticulum structural proteins in human left ventricular cardiomyocytes according to heart failure etiology and compared to a control (CNT) group. (**A**) Ribosomal receptor-binding protein (RRBP1) levels. (**B**) Immunofluorescence of RRBP1 protein. (**C**) Kinectin protein levels. (**D**) Immunofluorescence of kinectin. The values are normalized to GAPDH and finally to the CNT group. Values from the CNT group are set to 100. The data are expressed as means ± standard error of the mean (SEM) in optical density, arbitrary units (AU), of two independent experiments. *p<0.05, **p<0.01, ***p<0.001 *vs.* the CNT group. The bar represents 10 µm.

**Figure 4:**
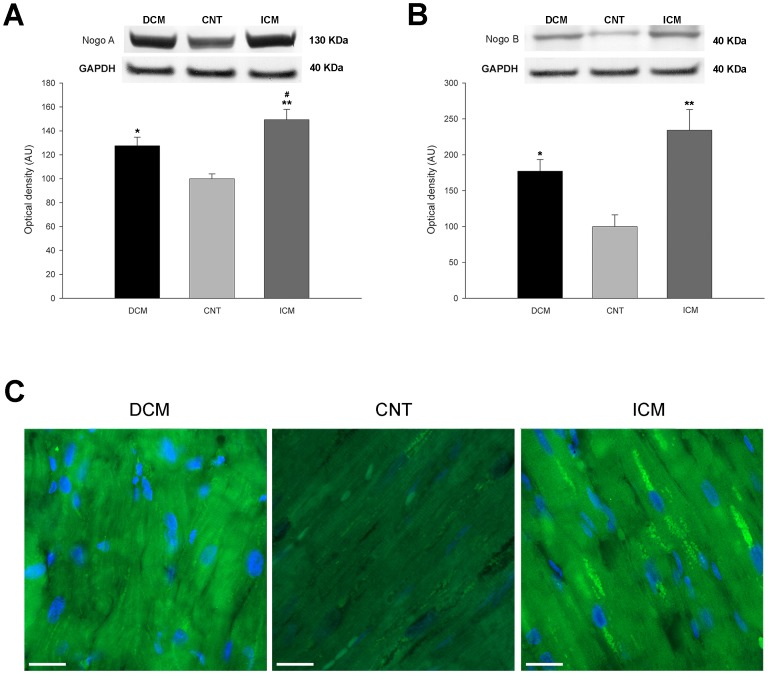
Western blot and immunofluorescence studies of Nogo A and B according to heart failure etiology and compared to a control (CNT) group. (**A**) Protein levels of Nogo A. (**B**) Protein levels of Nogo B. (**C**) Immunofluorescence of Nogo A+B. The values are normalized to GAPDH and finally to the CNT group. Values from the CNT group are set to 100. The data are expressed as means ± standard error of the mean (SEM) in optical density, arbitrary units (AU), of two independent experiments. DCM, dilated cardiomyopathy, ICM, ischemic cardiomyopathy. *p<0.01, **p<0.001 *vs.* the CNT group. #*p*<0.05 DCM *vs.* the ICM group. The bar represents 10 µm.

Furthermore, RRBP1 was significantly increased in the DCM and ICM groups compared with levels in the CNT group (311±208 *vs.* 100±54 AU, *p*<0.001, and 203±75 *vs.* 100±54 AU, *p*<0.01, respectively) (Fig. 3A) as well as kinectin (140±76 *vs.* 100±25 AU, *p*<0.05, and 161±94 *vs.* 100±25 AU, *p*<0.05, respectively) (Fig. 3C), Nogo A (128±33 *vs.* 100±13 AU, *p*<0.01, and 149±38 *vs.* 100±13 AU, *p*<0.001, respectively) (Fig. 4A), and Nogo B (177±76 *vs.* 100±52 AU, *p*<0.01, and 234±125 *vs.* 100±52 AU, *p*<0.001, respectively) (Fig. 4B). Our results also showed significant differences in Nogo A protein between the two pathological groups (128±33 *vs.* 149±38 AU, *p*<0.05) (Fig. 4A).

### Effect of HF on distribution of ER structural proteins

Additionally, we studied the distribution of some ER structural proteins using immunofluorescence. The results were consistent with the increased levels observed by Western blot. The high levels observed in DCM and ICM patients of RRBP1 (Fig. 3B), kinectin (Fig. 3D), and Nogo A+B proteins (Fig. 4C), were distributed in the same diffuse pattern in the cytoplasm of the cardiomyocytes in both, patients and CNT group.

### Relationship between stress and structural protein levels and with LV function

We further analyzed the relationships between proteins and we found significant relationships between the structural protein kinectin with Nogo A (*r* = 0.404, *p* = 0.016), Nogo B (*r* = −0.390, *p* = 0.017) and with Reticulon1 (*r* = 0.362, *p* = 0.042) in both the DCM and ICM groups; and between stress proteins GRP78 and XBP1 (*r* = −0.685, *p* = 0.001) in the DCM group. The structural protein RRBP1 and the stress protein XBP1 were related among them (*r* = 0.536, *p* = 0.022) in DCM.

**Figure 5:**
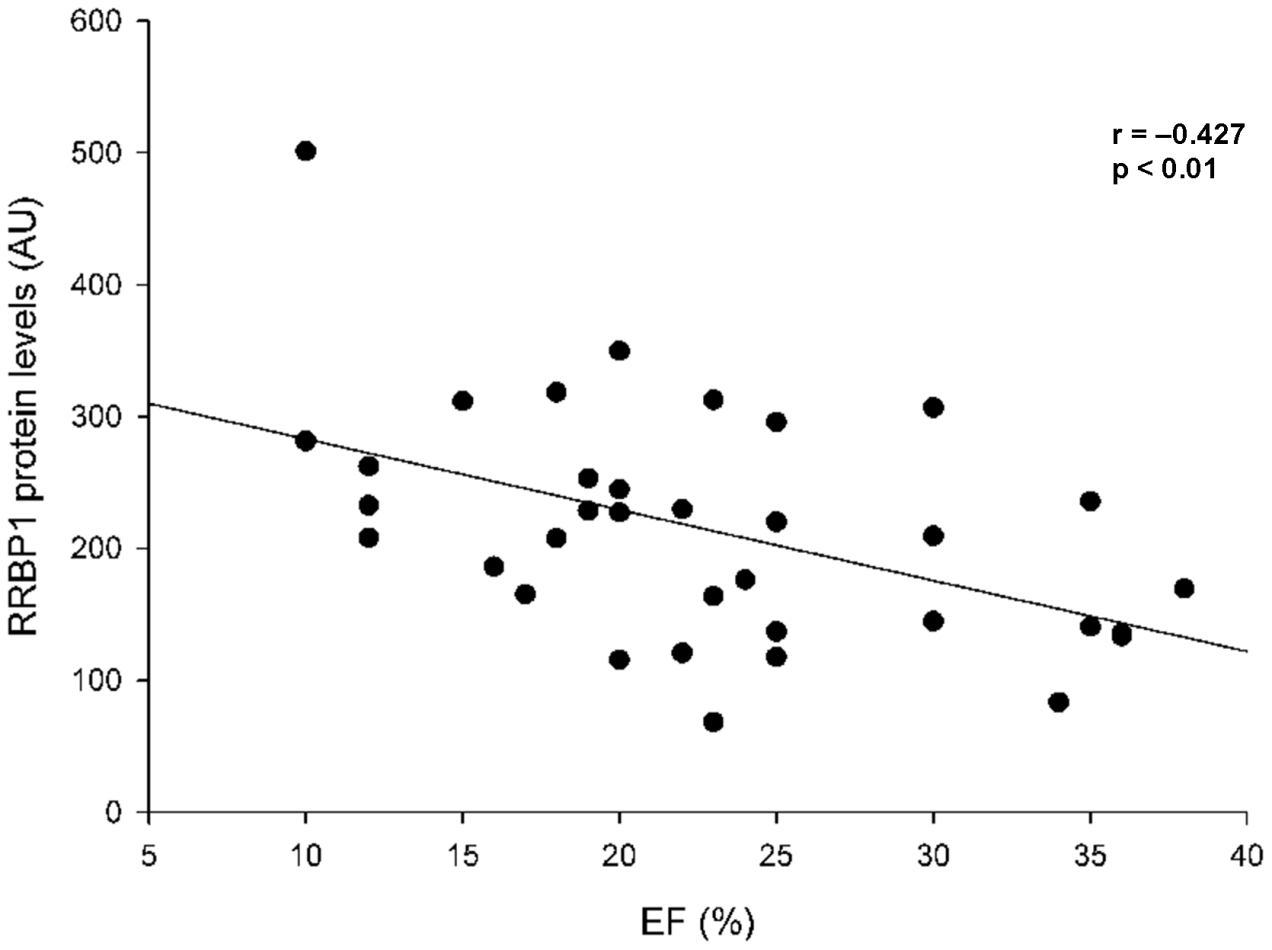
Scatter plots of correlation between RRBP1 and ejection fraction (EF) in dilated and ischemic cardiomyopathy human hearts. The values of RRBP1 are normalized to GAPDH.

Finally, we studied the relationship between these proteins and LV function. For 35 out of 42 samples, the LV function parameters were completely available. Of all the studied proteins, only RRBP1 was inversely related to EF (*r* = −0.464, *p* = 0.005) (Fig. 5) and fractional shortening (FS) (*r* = −0.463, *p* = 0.005) in DCM and ICM patients.

## Discussion

The present study provides new data into the ER stress process that occurs in HF patients [21], [22] and comprises not only alterations in stress-implicated molecules but also changes in ER structural proteins. We determined the levels of the most important proteins involved in these functions in the cardiac tissue of DCM and ICM patients. Our results revealed a general increase of these proteins in both pathological groups and some differences in protein levels between DCM and ICM. Furthermore, immunofluorescence images of structural proteins showed higher fluorescence intensity in DCM and ICM tissues compared with those in the CNT group, according to the observed protein expression levels.

### Transcriptional induction of ER stress proteins in HF

The UPR pathway is activated when ER stress is produced to re-establish the homeostasis of the ER. This adaptive response triggers the activation of three signaling pathways that include transcriptional induction of ER chaperones and folding catalysts, transcriptional activation of genes encoding components of ER-associated degradation, and general translational attenuation. However, if ER stress is prolonged or severe, apoptosis is induced to eliminate unhealthy cells [23]. In these pathways, three ER transmembrane sensors [IRE1, protein kinase-like ER kinase (PERK), and ATF6] initiate the response to manage accumulated unfolded proteins by facilitating the activation of the transcription factors XBP1, ATF6, ATF4 and eIF2α, which mediate ER stress response induction [24].

GRP78 is an ER chaperone that is used as a marker for ER stress [25], [26]. Under non-stressed conditions, GRP78 is bound to IRE1, PERK, and ATF6 and maintains them in an inactive state. In stress situations, GRP78 dissociates from these proteins, allowing their oligomerization to constitute an active form and triggering the UPR cascade [16], [27]. Our results show an upregulation of GRP78 protein in DCM and ICM patients compared with levels in CNT subjects, in presence of a decrease in IRE1, especially in ICM patients, as discussed later. Most studies report an upregulation of this protein in animal models of HF [25], [26], [28]. Studies of human DCM patients have also demonstrated upregulation of this protein [14] and, in addition, this protein has been proposed as a therapeutic target for reducing the effects of ER stress [29]. So, we can infer that in HF the increase observed in the level of GRP78 may be a consequence of UPR activation in the presence of ER stress.

IRE1 is a transmembrane serine/threonine protein kinase receptor activated by dissociation of GRP78, and phosphorylation, is the most fundamental ER stress sensor that inhibits protein synthesis, it has an endonuclease activity that specifically cleaves an alternative spliced mRNA encoding the transcription factor XBP1 [30], [31]. This transcription factor activates the transcription of genes involved in the ER stress response. IRE1 was downregulated in ICM patients in our work, and a similar tendency was found in the DCM group. We also analyzed the levels of the activated form of IRE1, p-IRE1, and we found that this protein was upregulated in DCM patients and the same tendency was shown in ICM. Interestingly, XBP1 protein displayed increased levels in the two pathological groups studied. The decreased levels observed in IRE1 and the increased levels of p-IRE1 are also consistent with the activation of the UPR mechanism, since this not implies an increase in the protein levels but also the activation is possible through dimerization and autophosphorylation [32]. XBP1 upregulation has been reported in studies analyzing hypoxic effects in mouse culture cardiomyocytes [21]. All of these findings are consistent with the XBP1 activation in the UPR to increase the transcription of ER stress response genes.

ATF6 is a ER transmembrane signaling protein that together with IRE1 and PERK are the major sensors of unfolded proteins of the ER. ATF6 is activated by dissociation of GRP78 and its translocation to the Golgi and to the nucleus [24], is responsible for transcriptional regulation of pro-survival genes following ER stress, inducing the expression of ER stress genes [16]. We found that ATF6 was upregulated in both DCM and ICM patients, demonstrating that this ER stress pathway is highly important to activate the response during HF. All these results evidence the strong activation of UPR mechanism in HF patients that implies alterations in the levels of the majority of ER stress proteins.

### ER structural protein levels in HF

Due to the ER stress process, is probably that a modification of the structure and shape of ER could occur in HF. The reticulons are a family of proteins that function mainly to stabilize the curvature of ER tubules [33]. In this work, we analyzed three members of this family, Reticulon 1 and two isoforms of Reticulon 4 (Nogo A and B). We found that Reticulon 1 is downregulated in DCM patients, whereas Nogo A and B were upregulated. Voeltz *et al.* have studied the effect of expression modification of reticulons *in vitro* and found that the downregulation of Reticulon 1 under stress conditions produces changes in the peripheral ER through the formation of membrane sheets; when the reticulons are overexpressed, tubule formation increases [34].

These changes in the protein levels of reticulons may alter the stabilization of the ER curvature, conferring it a different structure. Nevertheless, none studies have examined this relationship in heart disease; however the findings shown in this study strongly suggest that vital functions of the ER rely on its morphological integrity.

Other structural proteins analyzed were RRBP1 (also known as p180), kinectin, and CKAP4. A common feature of these proteins is their coiled-coil domains that form luminal bridges (in the case of CKAP4) and flat scaffolds (in RRPB1 and kinectin proteins). These proteins are implicated in shaping, cisternae stacking, and cytoskeletal interactions [35]. RRBP1 and kinectin are ER integral membrane proteins. The former plays a role in polysome assembly and therefore in protein biosynthesis as well as expansion of the Golgi complex [36]; also, it has been shown that is an inductor of membrane biogenesis [37], [38]. Kinectin is a receptor for kinesin, which is involved in cellular component transport along microtubules [39].

Both RRBP1 and kinectin were upregulated in the pathological groups studied. These results could indicate that modeling of the ER structure occurs as a possible consequence of UPR activation in ER stress conditions. Moreover, the upregulation of RRBP1 may influence the enhancement of protein synthesis necessary in some UPR pathways. In addition, we found a direct relationship between this protein and the stress protein XBP1 in the DCM group, giving evidence about the existence of a specific relationship between stress and structure.

Besides, we found a solid inverse relationship between RRBP1 and LV function parameters EF and FS. These parameters are related to the ventricular remodeling that occurs in HF patients. This ventricular remodeling implies the activation of several pathways including inflammation and fibrosis that need a production of their related proteins. RRBP1 has a relevant role in protein synthesis and particularly it has been shown that is a crucial step in cardiac remodeling throughout increasing procollagen synthesis [40], necessary to develop the fibrosis processes in maladaptive cardiac remodeling.

In addition, there is evidence supporting structural modifications under stress conditions. Tumarovs'ka *et al.* reported the dilation of ER cisternae in induced ER stress in isolated cardiomyocytes [41]. Taking into account these findings and altogether with the results obtained in our study, we conclude that alterations in various UPR molecules as a consequence of ER stress may influence the architecture of the ER and alter its structure since we have observed changes in some of the proteins involved in forming and organizing the ER.

### Study limitations

One limitation of this work is the inherent variability of the samples in disease etiology and treatment. However, to make our study etiologically homogenous, we chose DCM patients that reported no family history of the disease. Moreover, the patients selected in this study were on conventional therapy, and certain drugs may have influenced the levels of ER proteins. Furthermore, our tissue samples were confined to the transmural left ventricle apex, so our findings cannot be generalized to all layers and regions of the left ventricle. It is also noteworthy that this study was carried out using a high human sample size of both pathological and CNT hearts, making our results applicable for both DCM and ICM population.

## Conclusions

We report the existence of new alterations in proteins related to ER stress response and shaping in HF. We show a plausible effect of ER stress on ER structure in a suitable sample of human hearts with DCM and ICM. Patients with higher values of RRBP1, linked to procollagen production, had worse LV function. Our results show a novel relationship between stress and structure of the ER and may be used to find specific therapeutic targets.
